# Effect of an implementation support program aimed at an evidence-based physical healthcare model for patients with severe mental illness

**DOI:** 10.1186/s12888-026-08261-0

**Published:** 2026-06-11

**Authors:** Stine Larsen, Jūratė Šaltytė Benth, Tordis Sørensen Høifødt, Torleif Ruud, Anne Høye

**Affiliations:** 1 Department of Clinical Medicine, UiT - The Arctic University of Norway, Tromsø, Norway; 2 Division of Mental Health and Substance Abuse, University Hospital of North Norway, Tromsø, Norway; 3 Institute of Clinical Medicine, Campus Ahus, University of Oslo, Oslo, Norway; 4 Health Services Research Unit, Akershus University Hospital, Lørenskog, Norway; 5 Division of Mental Health Services, Akershus University Hospital, Lørenskog, Norway

**Keywords:** Severe mental illness, Schizophrenia, Cardiometabolic risk, Physical health care, Evidence-based practices, Fidelity scales

## Abstract

**Background:**

Patients with severe mental illness (SMI) experience higher rates of morbidity and mortality from physical conditions, particularly cardiovascular disease, due to modifiable risk factors and inadequate healthcare. This study aimed to assess whether an implementation support program, directed at a model for evidence-based physical healthcare, effectively improved fidelity and subsequently patient outcomes compared to no such support.

**Methods:**

In this multicentre study on the implementation of national clinical guidelines for physical health care for patients with SMI, 26 clinical sites within specialist mental health services in Norway participated between June 2016 to April 2018. Thirteen sites received implementation support, while the remaining thirteen did not. Fidelity to the evidence-based model for physical healthcare was measured using The Physical Health Care Fidelity Scale. Fidelity assessments and data from 230 patients across these sites were collected at baseline, 12 months, and 18 months. Changes in fidelity scores and outcome variables (cardiovascular risk factors and received evidence-based treatment of physical healthcare) from baseline to follow-up were analyzed using generalized linear mixed models.

**Results:**

Clinical sites showed consistently low mean fidelity scores, but those with implementation support had greater improvements (mean increase of 0.9 vs. 0.2). Higher fidelity was associated with increased odds for patients receiving physical activity and somatic healthcare interventions (OR 1.73, 95% CI (1.10; 2.72) and OR 1.52, 95% CI (1.07; 2.17), respectively). Smoking cessation interventions significantly increased at intervention sites from baseline to 12 months (OR 4.32, 95% CI (1.28; 14.57)), and by 18 months, somatic healthcare interventions were more common at sites with implementation support (OR 6.13, 95% CI (1.84; 20.37)). Cardiovascular risk factors in participants were not associated with fidelity.

**Conclusion:**

Clinical sites receiving extensive implementation support demonstrated promising improvements in overall fidelity scores compared to those that did not. However, no associations were identified between fidelity and patient outcomes. Fidelity scores remained low across all included sites, reflecting a generally limited implementation of evidence-based physical healthcare. This highlights the need to employ standardized fidelity scale measures to empower the specialist mental health system to identify areas requiring improvement and guide future interventions.

**Trial registration:**

The trial was retrospectively registered in ClinicalTrials.gov 31.08.2017, ID: NCT03271242.

**Supplementary Information:**

The online version contains supplementary material available at 10.1186/s12888-026-08261-0.

## Background

Patients with severe mental illness (SMI) have higher morbidity and mortality rates from physical illnesses compared to the general population, primarily due to inadequate prevention and treatment of cardiovascular diseases [1–5]. Individuals with SMI are predisposed to metabolic syndrome, characterised by hypertension, dyslipidemia, glucose intolerance and obesity – all established risk factors for diabetes and cardiovascular disease. Additionally, they exhibit higher rates of lifestyle risk factors, with smoking having the most severe health consequences [6–10]. They exhibit sedentary behaviour with less physical activity compared to individuals without mental illness, and their diets often do not align with dietary guidelines, featuring a higher intake of calories and sodium and a lower consumption of nutrient-rich foods [11, 12]. Essential treatment with antipsychotic medication has adverse effects, further exacerbating the unfavourable health outcomes they experience [13, 14]. SMI patients acquire these modifiable risk factors for cardiovascular diseases earlier in life than the general population, underscoring the critical need for early detection and management to prevent morbidity and premature mortality [15–18]. Furthermore, their dental health is negatively affected by their psychiatric illness, medication use, and overall lifestyle, resulting in poor dental health, which can further impact their overall health and well-being [9, 19, 20].

As a result, evidence-based practices and interventions for physical and dental health care have been developed and incorporated into clinical guidelines with the goal of improving healthcare for this patient group [21–23]. Unfortunately, there is currently limited knowledge regarding the extent to which the healthcare system has successfully implemented these guidelines into routine clinical practice as recommended. Even less is known about the implementation process itself [24, 25]. Consequently, there is scarce knowledge about how implementation support affects the degree of implementation and whether implementing clinical guidelines and evidence-based practice ultimately improves patient clinical outcomes and satisfaction [26–28]. Understanding these relationships is crucial for optimizing healthcare delivery and improving the overall well-being of this vulnerable population.

Studies have shown that even when guidelines are available, adherence to recommendations for physical health monitoring and intervention in mental health settings remains suboptimal, leaving cardiometabolic risk and disease undiagnosed and untreated [29]. To address this, a Norwegian multicentre implementation study was initiated with the goal of investigating the implementation of evidence-based practices, including physical health care, for the treatment of psychoses. Due to the lack of existing fidelity scales to measure evidence-based interventions for physical healthcare, the research group defined a cohesive and comprehensive model of physical healthcare for patients with SMI and developed the Physical Health Care Fidelity Scale which covers these evidence-based interventions [30].

Baseline findings from the study focusing on physical health care revealed poor implementation of evidence-based practices in specialist psychiatric healthcare for the identification, prevention, and treatment of cardiovascular risk factors, despite their high prevalence [31]. The aim of the present study was to investigate whether the implementation of an evidence-based model for physical health care could be improved through an implementation support program compared to no such support. The hypothesis was that sites receiving implementation support would achieve higher fidelity to The Physical Health Care Fidelity Scale, which, in turn, would positively influence treatment ant patient outcomes.

## Material and methods

### Study setting

The present study is part of a Norwegian cluster-randomized multicentre research project on the implementation of national clinical guidelines for the treatment of psychosis, conducted across 39 mental healthcare sites within six health trusts [32]. All sites were part of specialist mental health services, encompassing both tertiary care (hospital units) and secondary care (community mental health centres with outpatient clinics, day units, mobile teams, and inpatient wards). All participating sites collaborated with primary care providers, such as general practitioners (GPs) and municipal mental healthcare services.

### Fidelity

From the national guidelines, four different evidence-based practices were defined in the project: physical healthcare, antipsychotic medication, family psychoeducation, and illness management and recovery. The selection was based on criteria considered core elements of psychosis treatment, aligning with high-evidence clinical guidelines. Furthermore, the chosen interventions required either existing fidelity scales or the capacity to develop them, ensuring that the necessary clinical competence could be achieved through targeted training.

Each participating site selected two of the four practices. Through pairwise randomization, each site was then assigned to receive implementation support for one practice (the experimental condition) and no implementation support for the other (the control condition). Fidelity measurements were conducted by pairs of trained fidelity assessors selected from a pool of 15 researchers, which included psychologists, psychiatrists, nurses and other health professionals. The pairing of assessors varied somewhat across sites and measurement points. These assessors were independent of the clinical staff and adhered to fidelity guidelines when conducting assessments at each time point.

### Physical healthcare

This article focuses exclusively on the evidence-based practice of physical health care. Among the participating sites, 26 selected physical healthcare as one of their practices. All 26 sites were already routinely delivering physical healthcare to patients with mental disorders. However, following the randomization process, 13 sites were assigned to receive extensive implementation support, while the remaining 13 sites did not receive such support for implementing evidence-based physical healthcare. Data collection, including fidelity assessments and patient data, was conducted at baseline and during follow-ups at 6, 12 and 18 months, spanning the period of June 2016 to March-April 2018. Systematic training and support were provided to the sites that were randomly assigned to receive implementation. The first component involved a one-day workshop led by national experts on physical healthcare for individuals with psychosis, which was attended by leaders and clinicians from each site. Additionally, each site received a “Toolkit of Physical Health Care”, developed by the research team. This toolkit included a detailed description of the evidence-based components of physical healthcare, along with their rationale and references, key literature, and presentations from the workshop. The second, and most critical, component of the implementation support was delivered by implementation trainers who helped sites identify and overcome factors facilitating or hindering the process, and building robust systems to support implementation. This training aimed to bolster positive perceptions, ultimately ensuring the success of the implementation effort. For the first six months, the trainers visited each site biweekly, and for the remaining 12 months, they conducted monthly visits.

As no established fidelity scale covered the physical health practice, The Physical Health Care Fidelity Scale was developed by the research group. They utilized standardized procedures for fidelity scale development, as described in detail in a previous article [30]. Five evidence-based components of physical health care were identified from research reviews: promoting and supporting physical fitness, monitoring cardiovascular risk factors and treating physical illnesses, promoting and supporting healthy diets, smoking cessation, and dental health. The fidelity scale and its components are briefly presented in Table 1. Under each component, intervention items were further defined with specific operational criteria and rating instructions for each of these core components. The full scale, presented in supplementary Table 1, contains a total of 17 items, each scored individually on a 5-point scale based on the number of criteria met. Scores range from 1 to 3 (indicating poor implementation), to 4 (indicating adequate fidelity), and to 5 (indicating full implementation). The 17 items are assessed using two approaches: Items 1–6 are based on semi-structured interviews with leaders and clinicians, as well as written policies and procedures from each site, while items 7–17 are based on site practices, with information derived from 10 randomly selected patient records covering the previous 3–6 months.

**Table 1:** The Physical Health Care Fidelity Scale

Component of evidence-based physical health care	Scale items No	Short item titles
Policy and procedures promoting and supporting physical fitness	1	Policy promoting physical fitness
	2	Practical help to physical activities
	7	Support for regular physical activities
Policy and procedures promoting and supporting healthy diet	3	Policy to supporting healthy diet
	10	Documented support for healthy diets
Policy and procedures monitoring cardiovascular risk factors and treating physical illnesses	6	Collaboration and communication with GP
	8	Monitoring of physical health conditions
	9	Documented collaboration with GP
	11	Monitoring BMI and waist circumference
	12	Assessment and treatment of obesity/malnutrition
	13	Assessment and treatment of hypertension
	14	Assessment and regulation of blood sugar
	15	Assessment and regulation of blood lipids
Policy and procedures promoting and supporting smoking cessation	4	Policy supporting smoking cessation
	16	Interventions for smoking cessation
Policy and procedures promoting and supporting dental and oral health	5	Policy for supporting dental health
	17	Monitoring of dental health

### Participants

A total of 325 patients from the 39 mental health care sites were recruited by clinicians involved in their care to the overall cluster-randomized multicentre study. Of these, 230 patients were affiliated with the 26 sites (13 intervention sites and 13 control sites) that had physical health care as one of their practices, thereby qualifying for programme fidelity scoring.

The eligibility criteria for participation were being over 16 years of age and having a diagnosis of schizophrenia spectrum disorder (ICD-10 F20-F29), bipolar spectrum disorders (ICD-10 F30-F33), or current use of antipsychotic medication. The only exclusion criteria were the inability to give informed consent or understand Norwegian. The recruitment period lasted from June 2016 to March 2017.

At all timepoints, both participants and the health professionals responsible for their care completed questionnaires and rating scales to collect sociodemographic and health status information. In cases of uncertainty, clinicians validated this information using medical records. Somatic measurements were conducted for each patient and included weight, height, waist circumference, and systolic and diastolic blood pressure.

### Treatment intervention measures

Five outcome measures related to treatment interventions received by patients in the last six months were added (clinician-reported: yes/no). Each of these measures represented recommended interventions provided to the individual participants in accordance with guidelines. These interventions corresponded to the five fidelity components of the fidelity scale: organized physical activity/fitness, training courses in diet/cooking/nutrition, monitoring and treatment of physical risk factors and illnesses, plans for smoking cessation or reduction, and dental and oral health care.

For the assessment of cardiometabolic risk, eight clinical patient outcome measures were included. Body mass index (BMI) and waist circumference were recorded, while daily smoking or snuff use, unhealthy diets and sedentary behaviour were self-reported by participants. Sedentary behaviour was defined as engaging in one or fewer sessions of physical activity per week. Diet assessments were conducted by clinicians based on variation in diet composition according to national dietary guidelines [33] and categorized as “very healthy”, “somewhat healthy”, “somewhat unhealthy” and “very unhealthy”. These categories were later combined into a dichotomized variable: “healthy” or “unhealthy”. Hypertension was defined as blood pressure > 140/90 mmHg based on measurements and/or a recorded diagnosis, while diabetes and lipid disorder were defined by recorded diagnoses. Reports of smoking or snuff use, as well as assessments of diet and physical activity, were not included in the 12-month questionnaires. Consequently, these outcome measures are missing from the 12-month follow-up.

Furthermore, we assessed six patient variables: age, sex, education (dichotomized as completed high school versus not), use of antipsychotics (any type and dosage: yes/no), illicit drug use in the past six months (yes/no), and psychiatric disease severity measured by The Clinical Global Impressions Severity Scale (CGI-S) [34]. Illicit drug use was assessed based on clinician-reported information, including diagnoses related to psychoactive substance use, frequency and extent of drug use, and associated problems. Age and CGI-S were included as continuous variables. For the use of antipsychotics, dosage information was frequently missing, particularly at follow-up, although usage itself remained largely unchanged. Therefore, we included only reported antipsychotic use at baseline for this variable, as we did for age and educational level.

Fidelity scores from each patient´s corresponding clinical site were included as a measure of current fidelity. Both the total fidelity scale and the five different components were included as the main fidelity covariates. This approach provided both a general measure and more specific fidelity measures for the different evidence-based components of physical health care.

The Physical Health Care Fidelity Scale scores was compared with the registered treatment interventions for patients at intervention sites versus control sites at baseline, 12-month, and 18-month follow-ups. Furthermore, fidelity was compared to cardiovascular risk factors among patients in both groups across all three time points.

### Statistical analyses

Variables were described using appropriate descriptive statistics, mean and standard deviation (SD) for continuous variables, and number and percentage for categorical variables. The intra-class correlation coefficient (ICC) was estimated to assess cluster effect at site-level for all outcome variables. Generalized linear mixed models (GLMMs) were estimated to evaluate changes in outcome variables. These models included random effects for sites whenever ICC indicated presence of cluster effect. Further, the models included fixed effects for time (coded as dummy variables), group, and the interaction between these two. To assess the association between outcome variables and fidelity, a fixed effect for fidelity was included. Models for the five variables for received treatment interventions and three cardiovascular risk factors were adjusted for pre-selected baseline covariates: age, sex, education, use of antipsychotics, use of illicit drugs past 6 months, and CGI-S. For other outcomes, only unadjusted associations were presented due to low power. Only cases with no missing values on covariates were included in the analyses. To adjust for possible bias due to excluded cases, inverse probability weights were generated and applied. The results of GLMMs were tabulated as regression coefficients (RCs) with standard errors (SEs). Whenever suitable, odds ratios (ORs) and means with 95% confidence intervals (CIs) were also reported when presenting the results. As post hoc analyses, the within-group changes and between-group differences as well as between-group differences in changes were derived. Results with p-values < 0.05 were considered statistically significant. The analyses were performed in STATA v.17.0.

### Ethical considerations

Approval of research ethics was obtained from the Regional Committees for Medical and Health Research Ethics South-East Norway (REK 2015/2169) and each health trusts data protection officer. The principles in the Declaration of Helsinki were followed.

## Results

### Fidelity

**Fig. 1:**
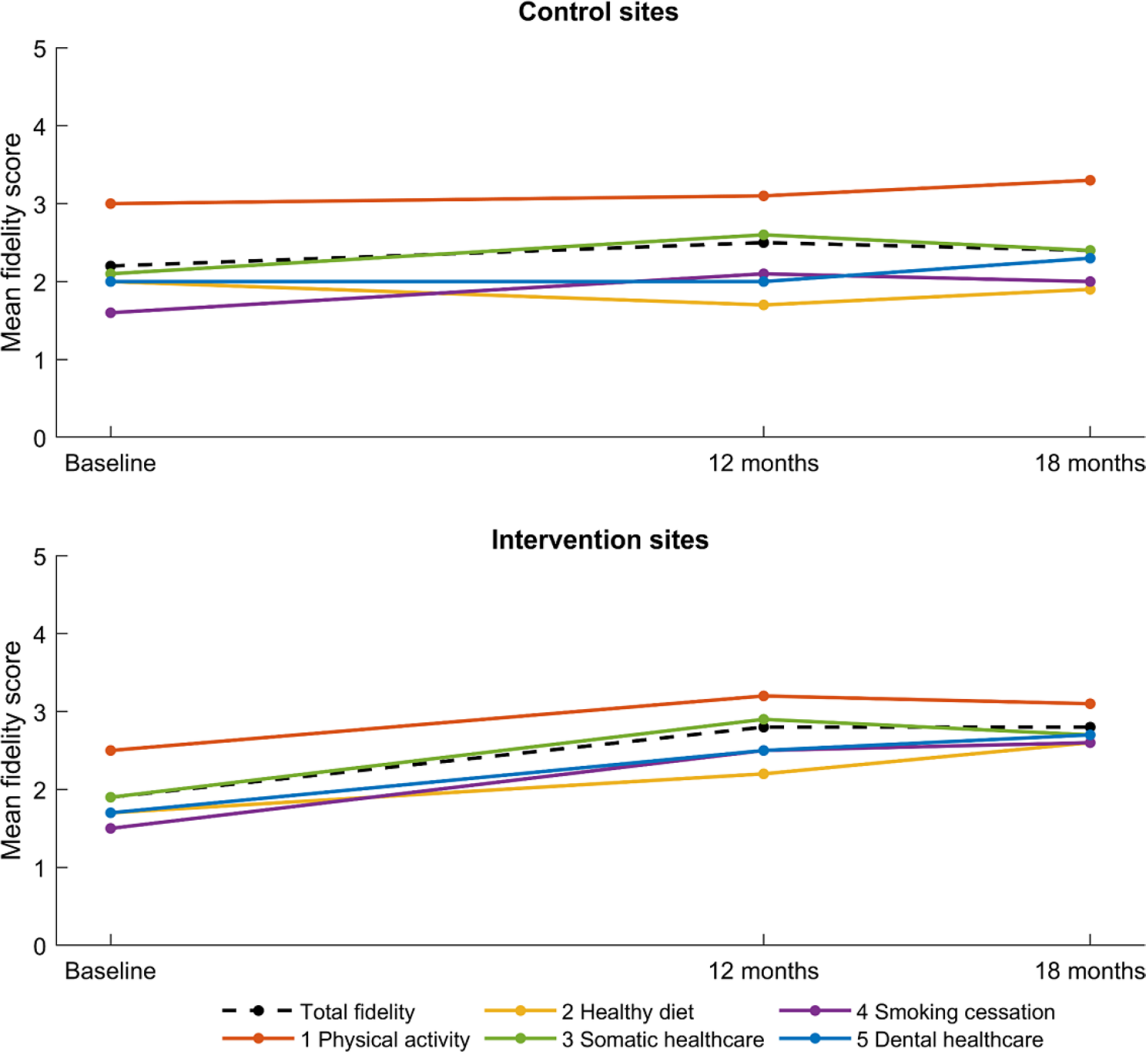
Mean fidelity score in the five components and total fidelity at baseline, and 12- and 18-month follow-up

Figure 1 presents the fidelity measurements at all sites (with exact values presented in supplementary Table 2), which were overall low, indicating poor implementation of recommended physical health care. At baseline, the 13 sites randomized to the control setting exhibited higher mean (SD) fidelity scores for both the total scale (2.2 (0.4) vs. 1.9 (0.5)) and its five different components compared to the 13 sites in the intervention setting. Although overall fidelity at all measurement points did not exceed 3.0 – and thus remained well below the threshold for adequate fidelity (fidelity score 4.0) – there was a clear positive trend in fidelity scores. This improvement is particularly evident for the intervention sites, which demonstrated a substantial mean fidelity increase to 2.8 (SD 0.6), compared to a modest mean increase to 2.4 (SD 0.4) in the control sites. Specifically, at the intervention sites, all five fidelity scale components showed increased scores, ranging from 0.6 to 1.1 from baseline to 18 months.

### Participants

The demographic and clinical characteristics of the patients at baseline and 12- and 18-month follow-up are presented in Table 2. At baseline, there were several notable differences between the control and intervention group: controls were slightly older, had a higher proportion of women and individuals with higher education, and reported a more frequent use of antipsychotics and illicit substances. Furthermore, their mean CGI-S score indicated mild disease severity, in contrast to participants receiving implementation support, whose mean CGI-S score reflected moderate disease severity. Table 2 also provides details on the distribution of cardiometabolic risk factors among the participants, with daily smoking and/or snuff use being the most prevalent at approximately 60%. The mean BMI in both groups indicated overweight, and 20–30% of participants presented with hypertension, sedentary behaviour and unhealthy diets. After adjusting for predefined covariates, no significant differences in cardiometabolic risk factors were observed between the two groups at baseline (displayed in Table 4 and supplementary Table 4).

**Table 2:** Demographic and clinical characteristics including cardiometabolic risk factors at baseline, and 12- and 18-month follow-up

Variables	Baseline		12 months		18 months	
	Control (*n* = 122)	Intervention (*n* = 108)	Control (*n* = 122)	Intervention (*n* = 108)	Control (*n* = 122)	Intervention (*n* = 108)
*Adjustment variables*						
Age, mean (SD) Gender, women, k/n (%) Higher education, yes, k/n (%) Use of antipsychotics, yes, k/n (%)	39.9 (12.4) 46/122 (37.7) 83/118 (70.3) 115/121(95.0)	37.9 (13.2) 44/108 (40.7) 71/105 (67.6) 98/105 (93.3)				
Use of drugs, yes, k/n (%)	46/121 (38.0)	34/106 (32.1)	8/37 (21.6)	4/37 (10.8)	9/63 (14.3)	5/41 (12.2)
CGI						
n	120	102	62	38	37	35
mean (SD)	3.7 (1.4)	4.5 (1.3)	3.3 (1.4)	3.9 (1.5)	3.3 (1.4)	4.4 (1.2)

***Outcome variables – cardiovascular risk factors***						
BMI						
n	86	85	43	30	27	29
mean (SD)	28.9 (5.6)	29.1 (7.0)	28.5 (5.8)	30.4 (7.5)	27.3 (5.3)	30.3 (7.6)
Waist width						
n mean (SD) Daily smoking, yes, k/n (%) Hypertension, yes, k/n (%) Diabetes, yes, k/n (%) Lipid disorder, yes, k/n (%) Unhealthy diets, yes, k/n (%) Sedentary behaviour, yes, k/n (%)	42 102.8 (15.9) 69/122 (56.6) 25/110 (22.7) 11/110 (10.0) 12/108 (11.1) 27/121 (22.3) 37/122 (30.3)	45 94.3 (15.3) 63/105 (60.0) 20/101 (19.8) 13/96 (13.5) 10/95 (10.5) 26/93 (28.0) 21/105 (20.0)	20 100.6 (11.8) 7/60 (11.7) 10/60 (16.7) 10/60 (16.7)	14 96.2 (25.7) 2/34 (5.9) 3/32 (9.4) 3/32 (9.4)	18 99.4 (16.0) 14/38 (36.8) 2/35 (5.7) 3/34 (8.8) 1/33 (3.0) 11/37 (29.7) 9/37 (24.3)	13 104.5 (16.6) 15/37 (40.5) 1/30 (3.3) 4/30 (13.3) 2/27 (7.4) 14/37 (37.8) 14/38 (36.8)

**Table 3:** Results of adjusted generalized linear mixed models for received treatment interventions, *n* = 216

	Physical activity (n=199+94+68=361, ICC=6.6%)		Healthy diet (n=199+93+66=358, ICC=11.2%)		Somatic healthcare (n=198+91+68=357, ICC=3.5%*)		Smoking cessation (n=199+90+62=351, ICC=1.3%*)		Dental healthcare (n=199+94+67=360, ICC=11.5%)	
	RC (SE)	p-value	RC (SE)	p-value	RC (SE)	p-value	RC (SE)	p-value	RC (SE)	p-value
InterceptTime	-1.36 (1.04)	0.191	-2.09 (1.04)	0.043	-1.71 (0.69)	0.013	-1.19 (1.50)	0.428	-3.18 (0.92)	0.001
Baseline – ref.	0		0		0		0		0	
12 months	0.25 (0.30)	0.388	0.70 (0.40)	0.076	-0.11 (0.36)	0.766	0.68 (0.54)	0.207	0.17 (0.31)	0.577
18 months	-0.27 (0.43)	0.533	0.57 (0.50)	0.250	-0.61 (0.42)	0.147	-0.06 (0.81)	0.940	0.32 (0.44)	0.468
Group (No support – ref.)	0.34 (0.34)	0.322	0.36 (0.41)	0.373	0.36 (0.32)	0.260	-0.17 (0.56)	0.756	0.19 (0.32)	0.558
Time x Group										
12 months x Support	-0.79 (0.49)	0.114	-0.85 (0.64)	0.185	0.02 (0.53)	0.973	0.78 (0.85)	0.358	0.31 (0.52)	0.548
18 months x Support	-0.09 (0.59)	0.875	-0.67 (0.70)	0.336	1.45 (0.66)	**0.028**	1.58 (1.19)	0.184	0.63 (0.62)	0.305
Fidelity-1 Physical activity	0.55 (0.23)	**0.020**								
Fidelity-2 Healthy diet			0.33 (0.33)	0.323						
Fidelity-3 Somatic healthcare					0.42 (0.18)	**0.020**				
Fidelity-4 Smoking cessation							-0.88 (0.47)	0.063		
Fidelity-5 Dental healthcare									0.27 (0.34)	0.432
Age	-0.01 (0.01)	0.196	-0.02 (0.01)	0.170	0.01 (0.01)	0.253	0.01 (0.01)	0.601	0.03 (0.01)	0.004
Gender, women	-0.16 (0.27)	0.545	-0.23 (0.34)	0.492	-0.001 (0.28)	0.998	-0.06 (0.40)	0.887	0.11 (0.28)	0.692
Higher education	-0.10 (0.30)	0.728	-0.09 (0.33)	0.782	-0.03 (0.29)	0.929	-0.98 (0.42)	0.019	-0.22 (0.30)	0.470
Use of antipsychotics	-0.70 (0.60)	0.241	-0.25 (0.54)	0.646	0.71 (0.43)	0.102	-0.16 (0.70)	0.817	0.51 (0.47)	0.278
Use of illicit drugs past 6 m	-0.16 (0.31)	0.609	0.05 (0.34)	0.887	-0.05 (0.31)	0.866	0.66 (0.45)	0.139	-0.11 (0.31)	0.720
CGI	0.22 (0.10)	0.027	0.24 (0.12)	0.049	0.03 (0.09)	0.714	0.16 (0.14)	0.245	0.12 (0.10)	0.216

*not adjusted for unit-level

Sample size decreased considerably at 12- and 18 months compared to baseline due to dropouts from various reasons. Specifically, the number of clinician-completed questionnaires decreased by approximately 55% at 12 months and 65% at 18 months. Among the remaining participants, a smaller proportion reported illicit drug use. The overall disease severity score remained relatively stable throughout the study period; however, there was a trend indicating that the remaining patients exhibited a lower severity level. This is reflected in the CGI-S mean scores, which decreased from baseline to 12- and 18 months for the control group (3.7 (SD 1.4) to 3.3 (SD 1.4)) and from 4.5 (SD 1.3) to 3.9 (SD 1.5) to 4.4 (SD 1.2) for the intervention group. The high dropout rate was likely influenced by a greater disease burden, characterized by psychiatric symptoms and drug use.

**Fig. 2:**
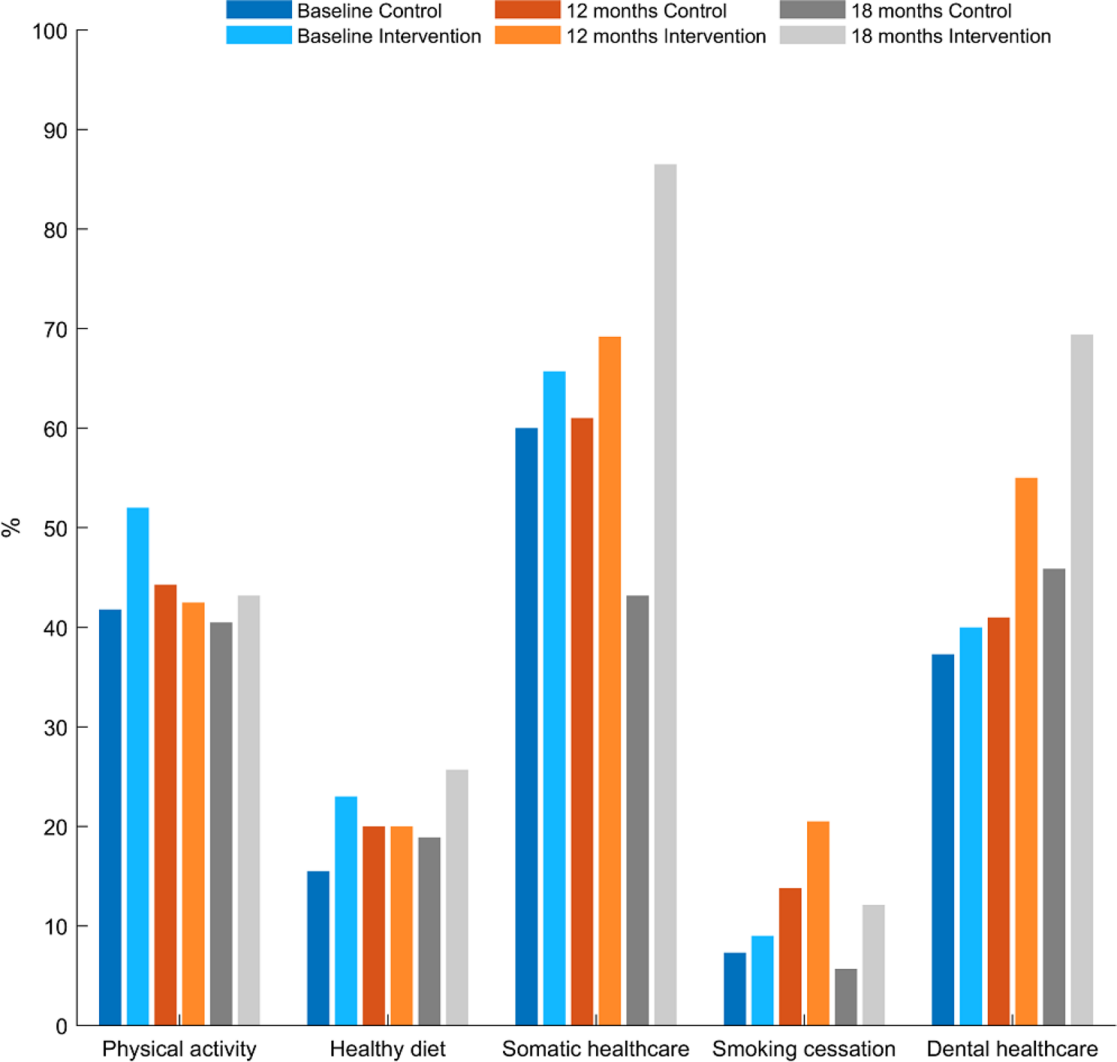
Percentage of patients receiving the different treatment interventions at baseline, and 12- and 18-month follow-up

Figure 2 illustrates the percentage of participants receiving treatment interventions corresponding to the five fidelity scale components at all measure points for both groups. At baseline, less than 10% of participants in both groups received smoking cessation interventions, while 15.5% in the control group versus 23.0% in the intervention group received interventions aimed at improving their diets. Dental healthcare interventions were provided to 37.3% of participants in the control setting and 40.0% in the intervention setting. Organized physical activity was accessed by 41.8% of participants in the control group and 52.0% in the intervention group. Somatic healthcare was the most common intervention, provided to 60.0% of participants in the control setting and 65.7% in the intervention setting. The figure also illustrates a sustained or improved level of interventions received by the participants according to clinicians at the intervention sites. Specifically, interventions aimed at somatic and dental healthcare reached 86.5% and 69.4%, respectively, at the 18-month follow-up. Only the intervention targeting physical activity shows an overall negative trend over time. In contrast, the participants assigned to the control sites demonstrated a negative trend at the end of follow-up for all interventions except dental healthcare.

### Fidelity and patient outcomes over time

According to the adjusted GLMM presented in Table 3 (results of unadjusted models are presented in supplementary Table 3), higher fidelity values were associated with higher odds of receiving treatment interventions aimed at physical activity and somatic healthcare (OR 1.73, 95% CI (1.10; 2.72), *p* = 0.020 and OR 1.52, 95% CI (1.07; 2.17), *p* = 0.020, respectively). According to the post hoc analysis, for the participants at the intervention sites, treatment interventions related to smoking cessation increased significantly from baseline to 12 months (OR 4.32, 95% CI (1.28; 14.57), *p* = 0.018). Additionally, at 18 months, the intervention group had significantly higher odds of receiving somatic healthcare compared to the control group (OR 6.13, 95% CI (1.84; 20.37), *p* = 0.003). There was also a significant difference in changes between the two groups from baseline to 18 months (OR 4.27, 95% CI (1.17; 15.63), *p* = 0.028), as well as from 12 to 18 months (OR 4.20, 95% CI (1.26; 13.97), *p* = 0.019), for somatic healthcare. Several significant associations were observed between the treatment interventions participants received and specific covariates. Illness severity, as measured by the CGI-S, was positively associated with interventions targeting physical activity (OR 1.25, 95% CI (1.02; 1.52), *p* = 0.027) and healthy diet (OR 1.27, 95% CI (1.00; 1.61), *p* = 0.049). A higher educational level was correlated with not receiving smoking cessation interventions (OR 0.38, 95% CI (0.16; 0.85), *p* = 0.019). Finally, higher age was associated with receiving dental healthcare (OR 1.03, 95% CI (1.01; 1.05), *p* = 0.004).

**Table 4:** Results of generalized linear mixed models for CV risk factors – BMI, Waist width and Smoking, *n* = 216

	BMI (n=165+61+47=273, ICC=13.0%)				Waist width (n=83+26+24=133, ICC=29.4%)				Smoking (n=213+0+71=284, ICC=0%*)			
	Unadjusted models		Adjusted model		Unadjusted models		Adjusted model		Unadjusted models		Adjusted model	
	RC (SE)	p-value	RC (SE)	p-value	RC (SE)	p-value	RC (SE)	p-value	RC (SE)	p-value	RC (SE)	p-value
InterceptTime	28.99 (0.61)	<0.001	24.66 (2.58)	<0.001	103.35(2.53)	<0.001	82.64 (7.73)	<0.001	0.26 (0.19)	0.157	0.12 (1.12)	0.918
Baseline – ref.	0		0		0		0		0		0	
12 months	-0.99 (0.77)	0.198	-0.16 (0.86)	0.851	-2.72 (2.84)	0.339	-6.97 (2.70)	**0.010**				
18 months	-1.50 (1.05)	0.154	-0.99 (1.14)	0.384	-3.84 (3.40)	0.260	-7.72 (2.97)	**0.009**	-0.83 (0.33)	0.012	-0.73 (0.34)	**0.032**
Group (No support – ref.)	-0.03 (0.99)	0.975	-0.80 (0.94)	0.393	-9.70 (3.37)	**0.004**	-6.06 (3.60)	0.092	0.12 (0.28)	0.666	0.19 (0.32)	0.564
Time x Group												
12 months x Support	2.83 (1.45)	0.051	2.15 (1.37)	0.117	9.21 (6.49)	0.156	9.04 (5.57)	0.104				
18 months x Support	2.79 (1.64)	0.089	2.39 (1.64)	0.146	16.15 (6.20)	**0.009**	15.84 (5.18)	**0.002**	0.04 (0.46)	0.929	0.02 (0.56)	0.969
Fidelity 3 Somatic healthcare	-0.76 (0.69)	0.267	-0.80 (0.65)	0.215	3.44 (2.16)	0.111	3.34 (2.16)	0.122				
Fidelity 4 Smoking cessation									0.16 (0.31)	0.612	0.04 (0.32)	0.893
Age	0.09 (0.04)	0.032	0.08 (0.04)	0.057	0.39 (0.14)	0.005	0.24 (0.16)	0.127	-0.006 (0.01)	0.578	0.006 (0.01)	0.661
Gender, women	-0.16 (1.14)	0.889	-1.71 (1.13)	0.131	1.93 (4.13)	0.640	0.29 (3.83)	0.940	-0.33 (0.29)	0.249	-0.33 (0.32)	0.301
Higher education	-1.27 (1.28)	0.322	-1.47 (1.22)	0.230	8.13 (4.33)	0.060	7.49 (4.22)	0.076	-0.94 (0.33)	0.004	-1.01 (0.34)	0.003
Use of antipsychotics	3.04 (1.38)	0.027	2.65 (1.66)	0.109	4.81 (3.68)	0.191	4.62 (5.93)	0.436	0.35 (0.59)	0.556	0.39 (0.64)	0.540
Use of illicit drugs	-2.91 (1.09)	0.007	-3.16 (1.25)	0.011	-8.31 (3.50)	0.018	-7.10 (3.80)	0.062	0.83 (0.31)	0.008	0.75 (0.33)	0.023
CGI	0.81 (0.37)	0.031	0.86 (0.39)	0.026	-0.02 (1.24)	0.990	-0.93 (1.05)	0.377	0.06 (0.10)	0.541	0.01 (0.11)	0.917

*not adjusted for unit-level

Results from both adjusted and unadjusted models for the CV risk factors BMI, waist circumference, and smoking are presented in Table 4. Detailed results of unadjusted models for hypertension, diabetes, lipid disorder, unhealthy diets, and sedentary behaviour are shown in supplementary Table 4. Fidelity was not associated with any of the eight cardiovascular risk factors. For BMI, diabetes, lipid disorder, and unhealthy diets, no changes were observed from baseline to follow-up, and no significant differences were found between the groups. However, after adjusting for covariates, a significant decrease in waist circumference was observed in the control group at 12 months (mean − 6.97, 95% CI (-12.25; -1.68), *p* = 0.010) and 18 months (mean − 7.72, 95% CI (-13.54; -1.91), *p* = 0.009). The change in waist circumference from baseline to 18 months was significantly different between the two groups (mean 15.84, 95% CI (5.69; 25.99), *p* = 0.002). In the control group, there was a significant reduction in smoking from baseline to 18 months (OR 0.48, 95% CI (0.25; 0.94), *p* = 0.032), as well as a significant reduction in odds for hypertension from baseline to 12 months (OR 0.22, 95% CI (0.05; 0.86), *p* = 0.030). Conversely, the intervention group showed a significant increase in odds for sedentary behaviour from baseline to 18 months (OR 2.29, 95% CI (1.03; 5.06), *p* = 0.041).

## Discussion

A key finding in the study is that, although control sites initially exhibited higher fidelity scores, sites receiving implementation support showed significantly greater improvement over time. Consequently, intervention sites ultimately achieved higher mean fidelity scores on the total scale, as well as across four specific components (cardiovascular risk factors and physical illnesses, healthy diets, smoking cessation, and dental health). Higher fidelity values were associated with higher odds of receiving treatment interventions aimed at physical activity and somatic healthcare. Additionally, after 18 months of implementation, participants at the intervention sites demonstrated a significantly higher exposure to treatment interventions regarding somatic healthcare compared to participants at the control sites, and participants at sites receiving implementation support experienced a significant increase in smoking cessation treatment interventions from baseline to 12 months. Conversely, no significant changes were noted in either group from baseline to 12- or 18 months for interventions targeting physical activity or diet, and the observed changes in interventions targeting dental healthcare were no longer significant after adjusting for covariates.

Despite the improvements in fidelity, particularly at intervention sites, the persistently low mean fidelity scores highlight the inadequate implementation of evidence-based physical healthcare practices. Even after 18 months of implementation support, the limited progress aligns with prior research emphasizing the inherent complexity of integrating evidence-based practices within healthcare systems [35, 36]. Somewhat surprisingly, individuals with lower symptom severity and higher educational levels were less likely to receive interventions targeting physical activity, healthy diet and smoking cessation. This may be due to a preconceived assumption that the ability for self-care and managing one’s own health is higher among these patients. Although it is known that health literacy is better among individuals with higher education [37, 38], such an assumption regarding patients with severe mental illness could contribute to a bias towards less focus on information and follow-up among patients with somewhat less severe mental symptoms and/or higher education.

In Norway, the physical healthcare of patients with SMI has traditionally been managed by GPs, while specialist mental health services have focused on psychiatric care. This division of responsibilities may have led to clinicians in specialist mental health settings lacking the necessary qualifications to provide adequate physical healthcare. Conversely, some GPs may face difficulties in comprehending and managing the psychiatric symptoms of these patients [39, 40]. Together, these challenges may impede the delivery of recommended physical healthcare, as unqualified practitioners may deliver education and interventions with lower fidelity [41]. Unlike somatic specialties, psychiatry often exhibits more negative attitudes towards and less familiarity with evidence-based medicine and clinical guidelines [42]. The psychiatric discipline also tends to exhibit greater resistance to fidelity measures [43]. Practitioners’ commitment and adherence are likely to be low if they do not believe in the intervention, perceive it as incompatible with their professional orientation, or consider it irrelevant to their patients [44–46].

Providing recommended physical healthcare for patients with SMI remains a significant challenge, largely due to an intricate interplay of multi-level barriers related to the intervention itself, the practitioners delivering it, the organizational context, and the participants receiving it [40, 47, 48]. Examples of these barriers include systemic issues like fragmented health services and incompatible IT systems; provider-level obstacles such as diagnostic overshadowing and low confidence in physical health skills; and patient intrinsic factors, including internalized stigma, cognitive deficits, and illness symptoms that impede help-seeking or treatment adherence. Such barriers likely contributed to the low fidelity observed in this study. Furthermore, fidelity was not positively associated with patient-related outcomes. In fact, after adjusting for covariates, the control setting demonstrated significant advantages regarding some cardiometabolic risk factors. Specifically, participants in the control group showed a significant decrease in waist circumference at both 12 and 18 months compared to baseline. They also exhibited a significant reduction in the odds of smoking by 18 months and a significant reduction in odds of hypertension by 12 months. Conversely, the intervention group experienced a significant increase in the odds of sedentary behaviour. These findings could be partially explained by the differences in disease severity - the mean CGI-S score in the control group at baseline indicated mild disease severity, whereas the intervention group had a mean CGI-S score reflecting moderate disease severity.

The Physical Health Care Fidelity Scale assesses a wide range of clinical elements and aspects of physical healthcare that require interventions based on complex, individualized assessments by practitioners. This is unlike some other fidelity scales, which primarily focus on structural elements of practice, such as resource availability. Structural scales are generally easier to implement and tend to achieve higher fidelity scores [49, 50]. Our prior baseline findings indicate that this discrepancy extends to individual fidelity items on the Physical Health Care Fidelity Scale [31]. Specifically, clinical sites demonstrated higher overall fidelity scores for policy-related items compared to practice-related items. Moreover, the intervention descriptions within the scale may have been vague or ambiguous, potentially leading practitioners to misinterpret the core components they were expected to deliver [45, 51]. This could be addressed by revising the Physical Health Care Fidelity Scale to provide more precise descriptions, thereby enhancing the scale`s usability in clinical settings.

### Strengths and limitations

A key strength of this study is the repeated fidelity measurements using a validated fidelity scale for physical healthcare. This approach provides a valuable opportunity to address the knowledge gap regarding how the healthcare system can improve implementation of physical healthcare for patients with severe mental illness.

However, the study design had several limitations that affected our ability to draw conclusions about potential relationships between the fidelity of evidence-based physical healthcare and patient outcomes. These limitations included small patient sample sizes at the clinical sites, a relatively high dropout rate over the study period, and the absence of a direct linkage between the included participants and the patient records used in the Practices subscale of the Physical Health Care Fidelity Scale. Additionally, the high attrition rates may have influenced the validity of the findings, as the participants who remained at 12 and 18 months may have systematically differed from those who dropped out earlier. This discrepancy could have contributed to the higher rates of treatment interventions observed at 12 at 18 months at the intervention sites compared to participants at the control sites. Furthermore, the low variation in fidelity scores made it challenging to identify associations between fidelity and clinical outcomes.

In our study, several clinicians expressed scepticism regarding their sites’ low fidelity scores, believing they had provided the recommended physical healthcare to patients, which the fidelity measures may have failed to capture. This discrepancy could have explanations beyond an actual lack of implementation. For instance, clinicians may have made adaptations to the interventions that were inconsistent with fidelity, potentially resulting in the omission of core components of the evidence-based practice [52, 53]. The implementation support process was thoroughly discussed in advance; however, we cannot rule out the possibility that there was some variation in execution across the different intervention sites. Additionally, we cannot exclude the possibility that physical healthcare, although delivered as intended, was not adequately recorded in patients’ medical records. To account for the potential impact of under-documentation – rather than a true lack of implementation – as a factor contributing to low fidelity scores, we compared site-specific fidelity assessments with clinicians’ evaluations of whether the included patients had received the evidence-based physical healthcare practices. The proportion of patients reported as receiving treatment interventions varied across the intervention and control sites, over time, and among the different evidence-based components of physical healthcare. While some evidence suggests that missing documentation may have contributed to the low fidelity scores in our study, we cannot rule out the possibility that patients received increased clinician attention to physical healthcare solely due to their participation in the study. It is also possible that clinicians’ self-reflection on their delivery and competence in physical healthcare led them to report more favourable adherence to evidence-based practices.

During the same period as our project, a separate national follow-up study was conducted to introduce the “Healthy Heart Tool”, also aimed at improving cardiovascular disease risk management in individuals with SMI [54]. This parallel initiative may have mitigated the potential effects of the implementation strategies in our study, thereby reducing the differences between the intervention and control sites. Additionally, the study’s 18 months implementation period was relatively short, as both behavioral changes and shifts in clinical practice typically require more time to yield measurable health outcomes. Given these limitations, it may be more appropriate to focus on the identification of cardiovascular risk factors and the initiation of medical interventions for these health issues, rather than solely on the outcomes themselves (e.g., waist circumference, weight, BMI, diabetes, hypertension). Despite identifying several factors that likely influenced the overall fidelity levels in our study, we have documented a significantly greater increase in fidelity to evidence-based physical healthcare at sites receiving implementation support compared to control sites.

## Conclusions

The implementation of evidence-based physical healthcare practices within the specialist psychiatric healthcare system showed promising improvements at sites receiving comprehensive implementation support compared to those that did not, with higher fidelity scores over time. However, the overall implementation of physical healthcare presented considerable challenges across the included sites, characterized by low fidelity scores and minimal variation, which hindered the ability to identify associations with patient outcomes. There is a need for more rigorous studies on clearly defined physical health care interventions. Furthermore, the Physical Health Care Scale can serve as a valuable tool to gain deeper insights into service delivery and identify areas for improvement, thereby increasing the likelihood of achieving desired patient outcomes and contributing to strengthening the evidence base for the intervention.

## Supplementary Information

Below is the link to the electronic supplementary material.

Supplementary Material 1

Supplementary Material 2

## Data Availability

Due to ongoing investigations the datasets generated and analysed during the current study is only available from the corresponding author on reasonable request.

## Abbreviations

SMI: Severe mental illness
GPs: General practitioners
ICD-10: International Classification of Diseases, 10th revision
BMI: Body mass index
CGI-S: The Clinical Global Impressions Severity Scale
SD: Standard deviation
ICC: Intra-class correlation coefficient
RC: Regression coefficient
SE: Standard error
ORs: Odds ratios
