# Supplementary material for: Effect of an implementation support program aimed at an evidence-based physical healthcare model for patients with severe mental illness
Source: BMC Psychiatry. 2026 Jun 11;26:637. doi: 10.1186/s12888-026-08261-0 (PMC13483698; doi:10.1186/s12888-026-08261-0)
Supplement: Supplementary file 2 [file 12888_2026_8261_MOESM2_ESM.docx]

**Supplementary Table 2. Treatment received and Fidelity scores at baseline, 12- months and 18-months follow-up**

| **Variables** | **Baseline** | | **12 months** | | **18 months** | |
| --- | --- | --- | --- | --- | --- | --- |
|  | **Control (n=122)** | **Intervention (n=108)** | **Control (n=122)** | **Intervention (n=108)** | **Control (n=122)** | **Intervention (n=108)** |
| ***Outcome variables – treatment received*** | | | | | | |
| Interventions promoting physical activity  n  Nothing in this period  Yes (from sporadic to systematically)  Interventions promoting healthy diets  n  Nothing in this period  Yes (from sporadic to systematically)  Interventions promoting somatic health care  n  Nothing in this period  Yes (from sporadic to systematically)  Interventions promoting smoking cessation  n  Nothing in this period  Yes (from sporadic to systematically)  Interventions promoting dental health care  n  Nothing in this period  Yes (from sporadic to systematically) | 110  64 (58.2)  46 (41.8)  110  93 (84.5)  17 (15.5)  110  44 (40.0)  66 (60.0)  110  102 (92.7)  8 (7.3)  110  69 (62.7)  41 (37.3) | 100  48 (48.0)  52 (52.0)  100  77 (77.0)  23 (23.0)  99  34 (34.3)  65 (65.7)  100  91 (91.0)  9 (9.0)  100  60 (60.0)  40 (40.0) | 61  34 (55.7)  27 (44.3)  60  48 (80.0)  12 (20.0)  59  23 (39.0)  36 (61.0)  58  50 (86.2)  8 (13.8)  61  36 (59.0)  25 (41.0) | 40  23 (57.5)  17 (42.5)  40  32 (80.0)  8 (20.0)  39  12 (30.8)  27 (69.2)  39  31 (79.5)  8 (20.5)  40  18 (45.0)  22 (55.0) | 37  22 (59.5)  15 (40.5)  37  30 (81.1)  7 (18.9)  37  21 (56.8)  16 (43.2)  35  33 (94.3)  2 (5.7)  37  20 (54.1)  17 (45.9) | 37  21 (56.8)  16 (43.2)  35  26 (74.3)  9 (25.7)  37  5 (13.5)  32 (86.5)  33  29 (87.9)  4 (12.1)  36  11 (30.6)  25 (69.4) |
| ***Covariates*** |  |  |  |  |  |  |
| Fidelity Total, mean (SD)  Fidelity component 1 – Physical activity, mean (SD)  Fidelity component 2 – Healthy diets, mean (SD)  Fidelity component 3 – Somatic healthcare, mean (SD)  Fidelity component 4 – Smoking cessation, mean (SD)  Fidelity component 5 – Dental healthcare, mean (SD) | 2.2 (0.4)  3.0 (0.4)  2.0 (0.5)  2.1 (0.6)  1.6 (0.5)  2.0 (0.2) | 1.9 (0.5)  2.5 (0.7)  1.7 (0.4)  1.9 (0.7)  1.5 (0.3)  1.7 (0.2) | 2.5 (0.4)  3.1 (0.4)  1.7 (0.4)  2.6 (0.7)  2.1 (0.4)  2.0 (0.4) | 2.8 (0.6)  3.2 (0.6)  2.2 (0.4)  2.9 (0.7)  2.5 (0.7)  2.5 (0.8) | 2.4 (0.4)  3.3 (0.3)  1.9 (0.4)  2.4 (0.6)  2.0 (0.2)  2.3 (0.5) | 2.8 (0.6)  3.1 (0.7)  2.6 (0.4)  2.7 (0.9)  2.6 (0.8)  2.7 (0.8) |

**Supplementary Table 3. Results of unadjusted generalized linear mixed models for received treatment interventions, n=216**

|  | **Physical activity (n=199+94+68=361, ICC=6.6%)** | | **Healthy diet (n=199+93+66=358, ICC=11.2%)** | | **Somatic healthcare (n=198+91+68=357, ICC=3.5%*)** | | **Smoking cessation (n=199+90+62=351, ICC=1.3%*)** | | **Dental healthcare (n=199+94+67=360, ICC=11.5%)** | |
| --- | --- | --- | --- | --- | --- | --- | --- | --- | --- | --- |
|  | RC (SE) | p-value | RC (SE) | p-value | RC (SE) | p-value | RC (SE) | p-value | RC (SE) | p-value |
| Intercept  Time  Baseline – ref.  12 months  18 months  Group (No support – ref.)  Time x Group  12 months x Support  18 months x Support | -0.35 (0.20)  0  0.24 (0.28)  -0.21 (0.41)  0.29 (0.29)  -0.53 (0.46)  -0.006 (0.56) | 0.080  0.382  0.613  0.319  0.245  0.992 | -1.66 (0.27)  0  0.42 (0.36)  0.34 (0.49)  0.50 (0.36)  -0.60 (0.57)  -0.31 (0.67) | <0.001  0.246  0.495  0.170  0.289  0.639 | 0.42 (0.20)  0  0.16 (0.32)  -0.48 (0.39)  0.20 (0.30)  0.11 (0.52)  1.65 (0.65) | 0.033  0.619  0.213  0.496  0.830  **0.012** | -2.51 (0.37)  0  0.81 (0.51)  -0.13 (0.83)  0.14 (0.52)  0.23 (0.72)  0.60 (1.05) | <0.001  0.111  0.872  0.784  0.784  0.569 | -0.58 (0.20)  0  0.18 (0.29)  0.40 (0.37)  0.12 (0.29)  0.54 (0.48)  0.92 (0.55) | 0.004  0.535  0.280  0.682  0.258  0.092 |
| Fidelity-1 Physical activity  Fidelity-2 Healthy diet  Fidelity-3 Somatic healthcare  Fidelity-4 Smoking cessation  Fidelity-5 Dental healthcare | 0.59 (0.23) | **0.009** | -2.77 (0.78) | 0.126 | 0.44 (0.18) | **0.014** | -0.68 (0.44) | 0.116 | 0.27 (0.31) | 0.393 |
| Age  Gender, women  Higher education  Use of antipsychotics  Use of illicit drugs past 6 m  CGI | -0.02 (0.01)  -0.09 (0.26)  -0.24 (0.27)  -0.69 (0.55)  0.12 (0.29)  0.17 (0.10) | 0.063  0.721  0.383  0.214  0.674  0.095 | -0.02 (0.01)  -0.23 (0.31)  -0.21 (0.31)  -0.22 (0.55)  0.35 (0.34)  0.21 (0.11) | 0.100  0.461  0.490  0.689  0.305  0.056 | 0.02 (0.01)  0.09 (0.26)  0.01 (0.28)  0.85 (0.43)  -0.002 (0.30)  0.06 (0.09) | 0.103  0.725  0.966  0.047  0.994  0.512 | 0.003 (0.01)  -0.03 (0.39)  -0.83 (0.41)  0.21 (0.63)  0.54 (0.42)  0.19 (0.15) | 0.823  0.930  0.042  0.736  0.204  0.186 | 0.03 (0.01)  0.28 (0.26)  -0.09 (0.29)  0.84 (0.53)  -0.24 (0.29)  0.15 (0.09) | 0.001  0.285  0.745  0.114  0.416  0.102 |

*not adjusted for unit-level

**Supplementary Table 4. Results of adjusted generalized linear mixed models for CV risk factors – Hypertension, Diabetes, Lipid disorders, Unhealthy diets, Sedentary behavior, n=216.** Adjusted only for fidelity.

|  | **Hypertension (n=200+86+58=344, ICC=4.9%)** | | **Diabetes (n=198+86+58=342, ICC=12.0%)** | | **Lipid disorder (n=195+87+54=336, ICC=7.8%)** | | **Unhealthy diets (n=204+0+70=274, ICC=0%*)** | | **Sedentary behavior (n=213+0+71=284, ICC=0%*)** | |
| --- | --- | --- | --- | --- | --- | --- | --- | --- | --- | --- |
|  | RC (SE) | p-value | RC (SE) | p-value | RC (SE) | p-value | RC (SE) | p-value | RC (SE) | p-value |
| Intercept  Time  Baseline – ref.  12 months  18 months  Group (No support – ref.)  Time x Group  12 months x Support  18 months x Support | -1.42 (0.51)  0  -0.80 (0.43)  -1.57 (0.71)  -0.22 (0.35)  -0.53 (0.82)  ne | <0.001  0.060  **0.027**  0.534  0.513  ne | -2.37 (0.68)  0  0.45 (0.29)  -0.07 (0.48)  0.21 (0.43)  -0.69 (0.67)  0.25 (0.79) | 0.001  0.117  0.887  0.628  0.307  0.751 | -2.47 (0.65)  0  0.24 (0.38)  -1.31 (0.94)  -0.12 (0.46)  -0.34 (0.83)  0.98 (1.29) | <0.001  0.529  0.163  0.803  0.679  0.448 | -1.42 (0.70)  0  0.39 (0.45)  0.39 (0.35)  -0.05 (0.72) | 0.043  0.388  0.264  0.941 | 0.30 (0.85)  0  -0.08 (0.41)  -0.76 (0.37)  1.08 (0.57) | 0.722  0.844  **0.041**  0.059 |
| Fidelity 1 Physical activity  Fidelity 2 Healthy diet  Fidelity 3 Somatic healthcare | 0.11 (0.22) | 0.615 | 0.10 (0.31) | 0.756 | 0.20 (0.26) | 0.432 | 0.06 (0.32) | 0.841 | -0.39 (0.28) | 0.159 |

*not adjusted for unit-level

ne = not estimable
